# Global Trends in Survival From Astrocytic Tumors in Adolescents and Young Adults: A Systematic Review

**DOI:** 10.1093/jncics/pkaa049

**Published:** 2020-06-10

**Authors:** Fabio Girardi, Claudia Allemani, Michel P Coleman

**Affiliations:** Cancer Survival Group, Non-Communicable Disease Epidemiology Department, London School of Hygiene and Tropical Medicine, London, UK

## Abstract

**Background:**

Brain tumors represent an important cause of cancer-related death in adolescents and young adults. Most are diagnosed in low-income and middle-income countries. We aimed to conduct the first, to our knowledge, systematic review of time trends and geographical variation in survival in this age group.

**Methods:**

We included observational studies describing population-based survival from astrocytic tumors in patients aged 15-39 years. We queried 6 electronic databases from database inception to December 31, 2019. This review is registered with PROSPERO, number CRD42018111981.

**Results:**

Among 5640 retrieved records, 20 studies fulfilled the inclusion criteria. All but 1 study focused on high-income countries. Five-year survival from astrocytoma (broad morphology group) mostly varied between 48.0% and 71.0% (1973-2004) without clear trends or geographic differences. Adolescents with astrocytoma had better outcomes than young adults, but survival values were similar when nonmalignant tumors were excluded. During 2002-2007, 5-year survival for World Health Organization grade I-II tumors was in the range of 72.6%-89.1% in England, Germany, and the United States but lower in Southeastern Europe (59.0%). Five-year survival for anaplastic astrocytoma varied between 39.6% and 55.4% (2002-2007). Five-year survival from glioblastoma was in the range of 14.2%-23.1% (1991-2009).

**Conclusions:**

Survival from astrocytic tumors remained somewhat steady over time, with little change between 1973 and 2009. Survival disparities were difficult to examine, because nearly all the studies were conducted in affluent countries. Studies often adopted the International Classification of Childhood Cancer, which, however, did not allow to accurately describe variation in survival. Larger studies are warranted, including underrepresented populations and providing more recent survival estimates.

Primary tumors of the central nervous system (CNS) are rare. In adolescents and young adults (15-39 years), the estimated world-standardized incidence rate was 15 new cases per million in 2018, ranging from 29 in Western Europe to 4.4 in Eastern Africa (1). Although uncommon, in 20- to 39-year-olds, CNS tumors ranked second among the leading causes of cancer-related deaths in countries with very high human development index (2). Adolescents and young adults (15-39 years) are patients with distinct needs, and services provided for children and older adults may not be adequate (3).

In adolescents and young adults, almost 80% of CNS tumors are diagnosed in low-income and middle-income countries (1). Where the burden of CNS tumors is highest, however, patients may encounter obstacles in being diagnosed and treated for their disease. For instance, access to radiotherapy is extremely unequal worldwide. Density of radiotherapy machines varies between 4.9 or more per million population in Western Europe, North America, Australia, and Japan and 0.4 per million in the rest of the world (4). The divide between the number of diagnoses and the availability of treatment facilities will inevitably translate to missed opportunities of care (and cure), years of life lost, and financial hardship in families where patients are the breadwinners.

Mortality is a key indicator in epidemiological surveillance, but it does not provide information on the course of the disease following a cancer diagnosis. By contrast, population-based survival incorporates the follow-up component and reflects the overall effectiveness of a health-care system in managing that cancer (5,6).

The CNS comprises brain, spinal cord, and meninges. Brain tumors are, by far, the most important group. In the third cycle of the CONCORD programme, broad disparities in survival emerged among more than 650 000 adults who were diagnosed with a primary brain tumor in 58 countries worldwide during 2000-2014. Age-standardized 5-year net survival for all brain tumor subtypes and all ages combined (15-99 years) ranged between 14.7% in Thailand and 42.2% in Croatia.

Brain tumor morphology is the most important predictor of clinical outcome. In patients aged 15-44 years, the European average 5-year relative survival during 2000-2007 was 14.2% for glioblastoma but 56.1% for lower grade astrocytic tumors (7).

For adolescents and young adults diagnosed with a given brain tumor subtype, it is currently not known how survival varies around the world and whether it has improved over time.

As age increases from childhood to early adulthood, the morphology distribution shifts progressively from a predominance of low grade gliomas (eg, pilocytic astrocytoma) to a higher proportion of more aggressive tumors. The use of the International Classification of Childhood Cancer (ICCC) has been often extended to adolescents and young adults (8,9), but in light of the differences in the morphology distribution, it is unclear whether alternative strategies should be adopted.

We aimed to address these questions by systematically synthesizing the scientific evidence pertaining to population-based survival from brain tumors in adolescents and young adults.

## Methods

This systematic review focused on prospective, observational studies presenting survival from brain tumors in adolescents and young adults.

We queried 6 electronic databases (Dissertation and Theses Global, Embase, MEDLINE, Open Grey, Scopus, and Web of Science) from database inception to December 31, 2019. Search strategies were specific to each database and included terms referring to 4 domains: disease, statistical method, study design, and outcome. A professional librarian at the London School of Hygiene and Tropical Medicine reviewed the search strategies (Supplementary Table 1, available online).

**Table 1.** Summary of the studies included in the systematic review^a^

Study	Countries	Completeness of ascertainment	Population coverage	Calendar period for incident cases and end of follow-up	Age span, y	Quality indicators	Reference classification	Estimator
Gatta et al., 2003 (16)	EUROCARE-3 consortium	Not specified	Regional and national	1990-1994, not specified	15-24	Proportion of microscopically verified tumors, exclusions, proportion of patients censored before 5 y, proportion of unspecified morphologies	ICD-O-3	Observed survival
Pearce et al., 2005 (17)	England	98%	Regional: northern region	1968-1997, not specified	15-24	Not specified	Not specified	Observed survival
Stiller et al., 2006 (18)	ACCIS	Not specified	Regional and national	1978-1997, 2001	15-19	Proportion of microscopically verified tumors, exclusions, proportion of unspecified morphologies	ICD-O-2	Observed survival
Desandes et al., 2007 (19)	France	Not specified	Regional (10%): 9 registries	1978-1997, 2002	15-24	Not specified	ICD-O-2	Observed survival
Linabery et al., 2008 (20)	United States	98%	Regional (14%): SEER 13	1975-1999, not specified	15-19	Proportion of microscopically verified tumors, proportion of patients lost to follow-up	ICD-O-3	Observed survival
Gatta et al., 2009 (21)	EUROCARE-4 consortium	Not specified	Regional and national	1995-2002, 2003	15-24	Proportion of microscopically verified tumors, exclusions, proportion of unspecified morphologies	ICD-O-3	Observed survival
Aben et al., 2012 (22)	Netherlands	95%	National	1989-2009, 2010	15-29	Exclusions	ICD-O-1,2,3	Relative survival
Carreira et al., 2012 (23)	Portugal	Not specified	Regional (30%): 5 districts	1997-2006, 2010	15-24	Not specified	ICD-O-3	Observed survival
Jung et al., 2012 (24)	South Korea	Not specified	National	1999-2004, 2009	20-44	Proportion of microscopically verified tumors	ICD-O-3	Observed survival
Thumma et al., 2012 (25)	United States	Not specified	Regional: SEER	1973-2008, not specified	20-39	Not specified	Not specified	Observed survival
Gondos et al., 2013 (26)	Germany, United States	Not specified	Germany: regional (41%); United States: regional (14%)	1997-2006, not specified	15-39	Exclusions	ICD-O-3	Relative survival
Nicholson et al., 2013 (27)	England	Not specified	Regional: Yorkshire	1990-2004, 2009	16-24	Not specified	Not specified	Observed survival
Ho et al., 2014 (28)	Netherlands	98%	National	1989-2010, not specified	18-40	Not specified	ICD-O-3	Observed survival
Smoll et al., 2014 (29)	United States	Not specified	Regional: SEER 18	2000-2006, not specified	16-39	Not specified	Not specified	Relative survival
Brodbelt et al., 2015 (30)	England	Not specified	National	2007-2011, not specified	20-44	Proportion of microscopically verified tumors	ICD-O-2	Relative survival
Narita et al., 2015 (31)	Japan	Not specified	Not specified	2001-2004, not specified	20-39	Not specified	Not specified	Observed survival
Visser et al., 2015 (7)	EUROCARE-5 consortium	Not specified	Regional and national	2000-2007, 2008	15-44	Proportion of microscopically verified tumors, proportion of unspecified morphologies	ICD-O-3	Relative survival
Trama et al., 2016 (32)	EUROCARE-5 consortium	Not specified	Regional and national (12%-100%)	2000-2007, 2008	15-39	Proportion of microscopically verified tumors, exclusions, proportion of lost to follow-up, proportion of unspecified morphologies	ICD-O-3	Relative survival
Georgakis et al., 2017 (33)	SEE Consortium, United States	Not specified	SEE consortium: regional and national (5%-100%); United States: regional (28%): SEER 18	2001-2009, 2016	15-39	Proportion of microscopically verified tumors, exclusions, proportion of unspecified morphologies	ICD-O-3	Observed survival
Ostrom et al., 2017 (34)	United States	Not specified	Regional (26%): SEER 18	2000-2014, not specified	15-39	Proportion of microscopically verified tumors	ICD-O-3	Relative survival

a ACCIS = Automated Childhood Cancer Information System Consortium (Denmark, Estonia, Finland, France, Germany, Hungary, Iceland, Italy, Netherlands, Slovakia, Slovenia, Spain, Switzerland, United Kingdom, Norway); EUROCARE-3 = EUROCARE-3 Consortium (Austria, Czech Republic, Denmark, England, Estonia, Finland, France, Germany, Iceland, Italy, Malta, Netherlands, Norway, Poland, Scotland, Slovakia, Slovenia, Spain, Sweden, Switzerland, Wales); EUROCARE-4 = EUROCARE-4 consortium: Austria, Belgium, Czech Republic, Denmark, England, Estonia, Finland, France, Germany, Iceland, Ireland, Italy, Malta, Netherlands, Northern Ireland, Norway, Poland, Portugal, Scotland, Slovakia, Slovenia, Spain, Sweden, Switzerland, Wales; EUROCARE-5 = EUROCARE-5 Consortium (Austria, Belgium, Bulgaria, Croatia, Czech Republic, Denmark, England, Estonia, Finland, France, Germany, Hungary, Iceland, Ireland, Italy, Latvia, Lithuania, Malta, Netherlands, Northern Ireland, Norway, Poland, Portugal, Scotland, Slovakia, Slovenia, Spain, Sweden, Switzerland, Wales); EUROCARE= European Cancer Registry based study on survival and care of cancer patients; ICD-O = International Classification of Diseases for Oncology; SEE = South-Eastern European Consortium (Belarus, Bulgaria, Croatia, Cyprus, Greece, Malta, Portugal, Romania, Serbia, Slovenia, Turkey, Ukraine); SEER = Surveillance, Epidemiology, and End Results Program.

There is no consensus on the definition of “young adults,” and in most studies the upper age boundary varied between 24 and 39 years. We adopted a comprehensive approach by including patients aged 15-39 years. However, studies including individuals who overlapped this age range were still eligible.

We extracted data from published reports. Eligible studies had to include survival estimates from primary data collected in population-based cancer registries. For a given country or region, hospital-based estimates were retained only if no population-based estimates were available.

Studies were eligible if they included estimates derived from a time-to-event analysis and survival probabilities up to at least 5 years. More specifically, survival probabilities had to be estimated as observed survival, relative survival, or net survival (10). These outcome measures do not require knowledge of the cause of death.

We did not put restrictions relating to language and we considered both published articles and grey literature, such as conference abstracts and statistical reports. However, because morphology classifications changed substantially after 1995, we did not include earlier reports. If a study did not clearly meet the eligibility criteria, we decided on inclusion or exclusion through discussion.

We were interested in both nonmalignant and malignant brain tumors. We focused on astrocytic tumors because data for rarer subtypes were too scanty to allow robust comparisons.

Because morphological groupings differed between studies, we combined similar definitions (eg, anaplastic astrocytoma and astrocytoma World Health Organization [WHO] grade III) under a common descriptor (Supplementary Table 2, available online) but, where possible, without combining morphologies with different clinical behavior (ie, WHO grade). Definitions sharing the same code in the International Classification of Diseases for Oncology (ICD-O) Third Edition were merged (11). Then we conducted a sensitivity analysis by regrouping morphologies according to the Surveillance Epidemiology and End Results Adolescents and Young Adults (SEER AYA) Site Recode to explore whether less granular categories were equally informative (12). SEER AYA Site Recode is based on the classification proposed by Birch and Barr for tumors diagnosed in adolescents and young adults (13,14). SEER AYA Site Recode subdivides astrocytic tumors into 3 categories: low grade tumors, glioblastoma plus anaplastic astrocytoma, and astrocytoma not otherwise specified (NOS) (12).

From each eligible study, we abstracted 5-year survival probabilities by morphology. When studies provided survival estimates for more than 1 calendar period or considered more than 1 age group (eg, patients aged 15-19 years and 20-24 years), each estimate was considered separately. Where available, we collected specifications on the reference classification used for morphology definitions, data quality indicators (eg, proportion of microscopically verified tumors, patients lost to follow-up, and poorly specified/unspecified morphologies), and completeness of ascertainment.

Calendar periods differed between studies, and their length also varied. Therefore, we have presented the results labeled with the middle year of the calendar period.

The systematic review is registered with PROSPERO, number CRD42018111981.

## Results

**Figure 1.**
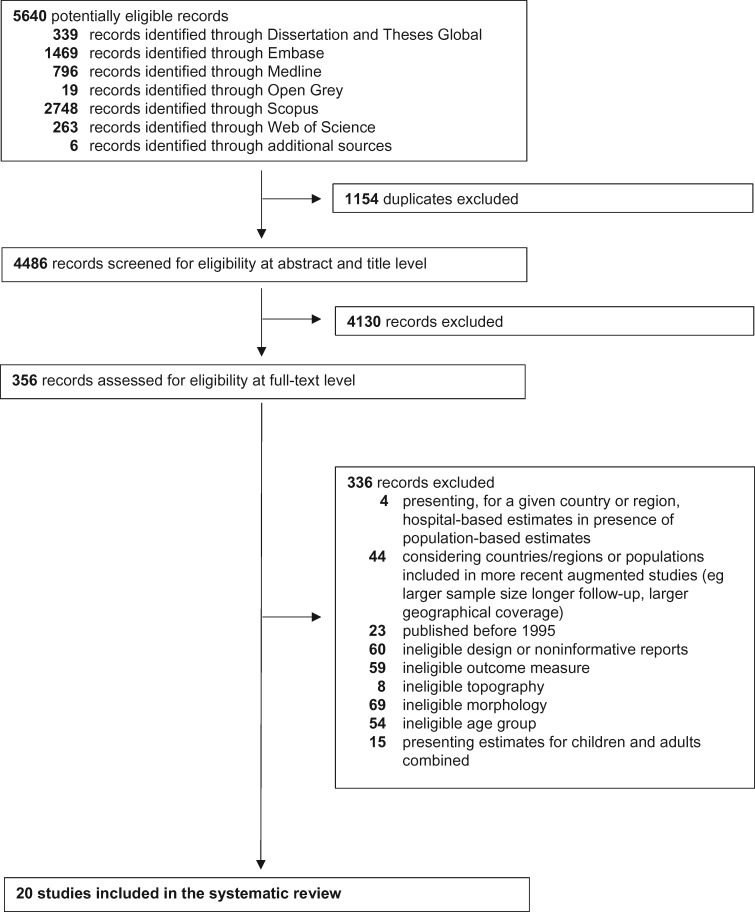
Preferred reporting items for Systematic Reviews and Meta-Analyses flowchart.

The database search yielded 5640 records. We screened these records for eligibility from the title and the abstract. We then assessed the full text of the remaining 356 publications for eligibility. This process followed the Preferred Reporting Items for Systematic Reviews and Meta-Analyses guidelines (Figure 1) (15). Twenty studies were included in the systematic review.

The calendar period for incident cases ranged from 1968 to 2014. Twelve studies (60%) were conducted in 1 or more European countries, 4 in the United States, 2 in Asia (South Korea and Japan), 1 comprised patients from the United States and Germany, and 1 international study was carried out in Europe but also included Cyprus and Turkey and the United States. Only 1 study included any data from middle-income countries (Table 1) (33).

Young adults were defined as individuals up to the age of 24 years in 6 studies, up to age 39 in 7 studies, and up to age 44 in 3 studies. Two studies adopted alternative age definitions of the upper boundary (29 or 40 years). The eligible studies collectively provided 75 survival estimates: 14 for adolescents only (15-19 years), 23 for young adults (20 years or more) only, and 38 estimates for adolescents and young adults combined (Table 1).

Eight of the 20 studies had regional population coverage, 4 were based on national registries, 7 were international studies drawing data from both regional and national registries, and in 1 study the information was not available (Table 1).

The completeness of ascertainment was specified in only 4 of the 20 studies (17,20,22,28). Twelve (60%) of the 20 studies provided details on data quality indicators: 2 studies specified only the criteria for exclusions (eg, diagnoses based on death certificate only or autopsy), and 10 reported at least the proportion of microscopically verified tumors (Table 1).

The proportion of microscopically verified tumors referred specifically to brain tumors in 4 studies (7,30,33,34), and in 5 the parameter was for all tumors combined (16,20,21,32,35). In the 2 international comparisons, the proportion of microscopically verified tumors varied between 57.2% and 96.4% (South-Eastern European [SEE] Consortium, plus the United States), and between 61.0% and 100% in the European Cancer Registry based study on survival and care of cancer patients (EUROCARE) 5 study, covering adolescents and young adults diagnosed during 1999-2007 in 27 European countries (7,33). One study comprised exclusively patients with microscopically verified tumors (Supplementary Table 3, available online) (24).

Five studies did not clarify the reference classification. In the remaining 15 studies, the second or third editions of ICD-O were the reference classification (Table 1) (11,36).

Ten of the 20 eligible studies grouped all astrocytic morphologies under the broad definition “astrocytoma,” but 2 of these studies did not clarify the behavior of eligible tumors (only malignant, or both malignant and nonmalignant) (Supplementary Table 3, available online) (23,27). Ten studies considered either subgroups (ie, low grade astrocytoma or high grade astrocytoma) or single morphologies (eg, diffuse astrocytoma, glioblastoma).

In 13 studies (65%), the outcome measure was observed survival (ie, all-cause survival), and in 7 studies it was relative survival (Table 1).

**Figure 2.**
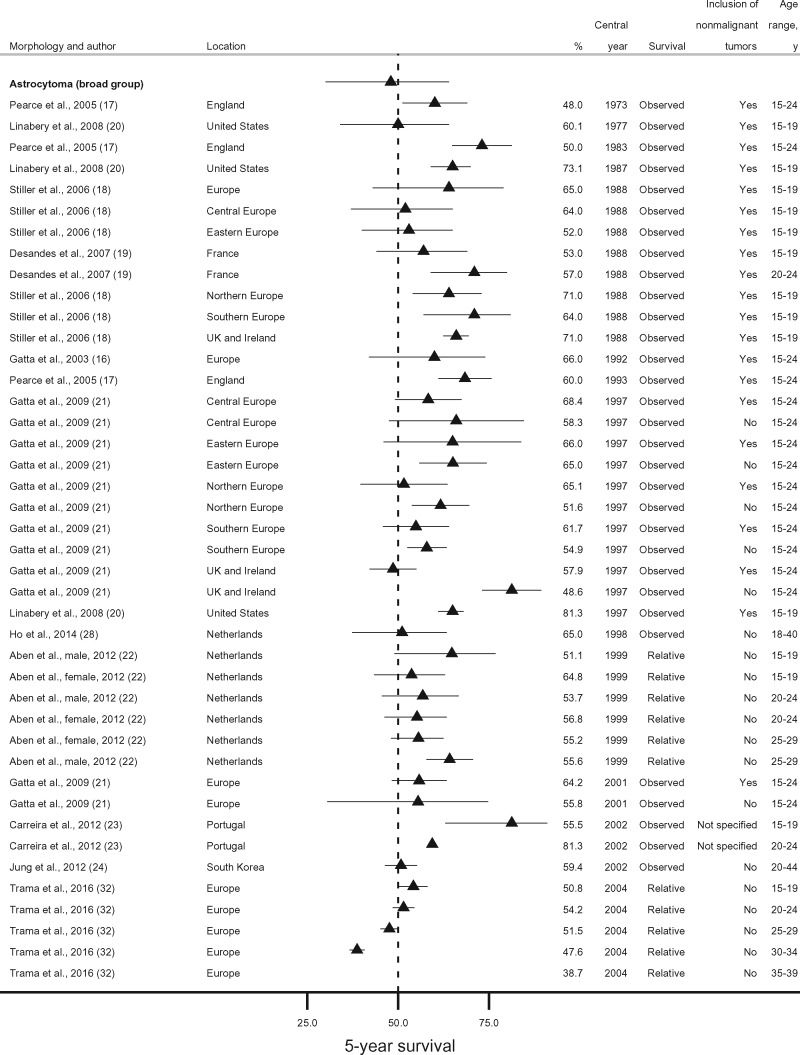
Five-year survival (percentage) from astrocytoma (broad morphology group). Error bars are 95% confidence intervals. For studies providing only point survival estimates, confidence intervals are shown as (survival estimate ±1%).

For astrocytoma as a broad morphology group, survival estimates referred mostly to patients aged 15-24 years. No studies were available from low-income or middle-income countries. Nearly all estimates of 5-year survival fell within the range of 48.0%-71.0% during 1973-2004, with little variation in survival between countries or over time (16–19,21–24,28,32). In the only US study, however, 5-year survival was higher than in Europe: 73.1% vs 65.0% around 1988, and 81.3% vs 64.2% around 2000 (20). In the EUROCARE-5 study for diagnoses during 2000-2007, 5-year survival from astrocytoma was 50.8% in adolescents (15-19 years), similar (47.6%) in patients up to 34 years old, but lower (38.7%) in the 35- to 39-year age group (Figure 2) (32). One study from Portugal, however, found that 5-year survival from astrocytoma was remarkably higher in young adults (20-24 years) than in adolescents (81.3% vs 55.5%), but confidence intervals were wide and largely overlapping because of the very small study population (51 patients overall) (23).

**Figure 3.**
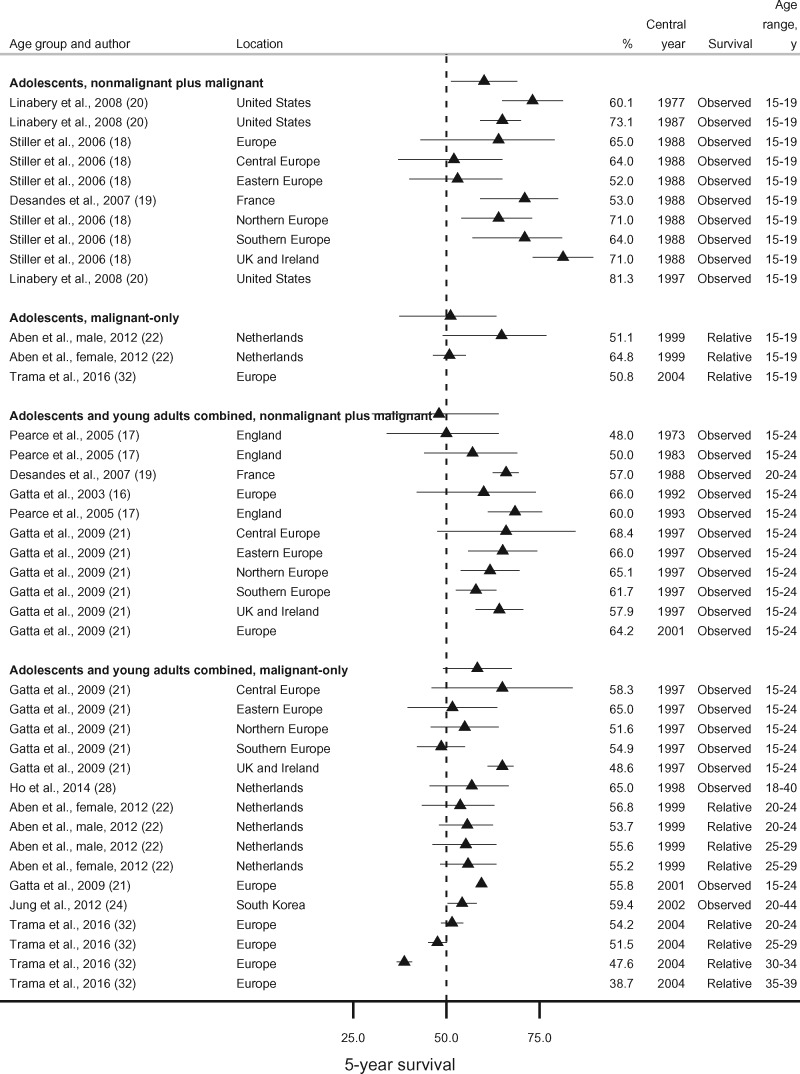
Five-year survival (percentage) from astrocytoma (broad morphology group) by age group (adolescents, or adolescents and young adults combined) and tumor behavior (nonmalignant plus malignant, or malignant only). Error bars are 95% confidence intervals. For studies providing only point survival estimates, confidence intervals are shown as (survival estimate ±1%).

Among the studies using the broad definition “astrocytoma,” we identified 4 possible combinations of age (adolescents [15-19 years] or adolescents and young adults combined [15-44 years]) and tumor behavior (all behaviors or malignant only). Five-year survival from nonmalignant and malignant astrocytoma combined was slightly higher in adolescents (15-19 years, 60.1%-81.3%) than in the broader age group (15-44 years, 48.0%-68.4%) except in Eastern Europe and France, where values were lower (52.0%) (18,19). Conversely, 5-year survival for malignant astrocytoma in adolescents was very similar to the values observed in adolescents and young adults combined (Figure 3).

**Figure 4.**
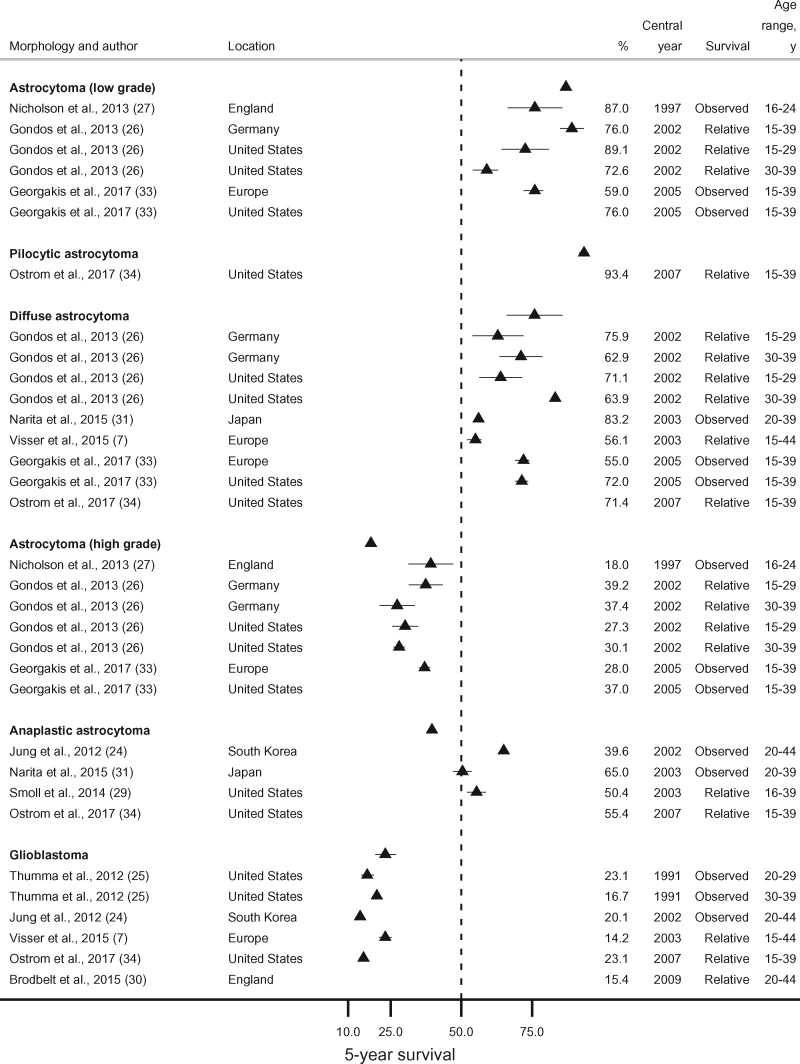
Five-year survival (percentage) from astrocytoma (low grade), pilocytic astrocytoma, diffuse astrocytoma, astrocytoma (high grade), anaplastic astrocytoma, and glioblastoma. Error bars are 95% confidence intervals. For studies providing only point survival estimates, confidence intervals are shown as (survival estimate ±1%).

Five-year survival from low grade astrocytoma (WHO grade I and II combined) was 87.0% or more for patients aged 16-29 years in England, Germany, and the United States (2002-2005). In the same countries, survival was lower (72.6%-76.0%) when individuals up to age 39 years were also included (26,27,33). In the SEE consortium, 5-year survival for patients diagnosed in 2005 was much lower (59.0%, 15- to 39-year-olds) (Figure 4) (33).

Five-year survival from high grade astrocytoma (WHO grade III and IV combined) was 18.0% in England in 1997 (27), whereas it varied between 27.3% and 39.2% in Germany, the United States, and the SEE consortium during 2002-2005 (Figure 4) (26,33).

Five-year survival from diffuse astrocytoma was in the range of 62.9%-75.9% in Germany and the United States during 2002-2007 (26,33,34). In the EUROCARE-5 consortium (27 European countries combined) and the SEE consortium (Southern and Eastern Europe), the average 5-year survival was 56.1% (2003) and 55.0% (2005), respectively (Figure 4) (7,33).

The survival probability at 5 years for South Korean patients diagnosed with anaplastic astrocytoma in 2002 was 39.6% (24). In the United States, 5-year survival was higher: 50.4% in 2003 and 55.4% in 2007 (Figure 4) (29,34).

Five-year survival from glioblastoma was in the range of 14.2%-23.1% in England, the EUROCARE-5 consortium, the United States, and South Korea without improvements in the 20 years between 1991 and 2009 (Figure 4) (7,24,25,30,34).

Last, studies were grouped according to SEER AYA Site Recode (12). Such system does not adopt the broad definition “astrocytoma,” so studies using this definition were excluded from the analysis. None of the studies focused solely on adolescents (15-19 years). Five-year survival from low grade astrocytic tumors was in the range of 71.4%-93.4% (26,27,31,33,34). Five-year survival from high grade astrocytic tumors varied between 14.2% and 55.4% (7,24–27,29,30,33,34). Five-year survival for astrocytoma NOS was in the range of 55.0%-75.9% (Supplementary Figure 1, available online) (7,26,33).

## Discussion

To our knowledge, this is the first systematic review summarizing international trends in survival from astrocytic tumors in adolescents and young adults (15-39 years).

Outcomes remained somewhat steady over time, with little change over the 35 years between 1973 and 2009. Five-year survival for all astrocytic tumors combined was mostly in the range of 48.0%-71.0%. Survival was much lower in studies including only patients with malignant astrocytoma or considering broader age groups.

Five-year survival was in the range of 55.0%-75.9% for diffuse astrocytoma, but it rose up to 89.1% when WHO grade I and II tumors were combined. The survival probability at 5 years was 23.1% or less for glioblastoma, in the range of 39.6%-55.4% for anaplastic astrocytoma, and mostly between 27.3% and 39.2% when the 2 morphologies were jointly considered. For a given morphology, older patients experienced poorer outcomes.

Nearly all the studies were conducted in high-income countries, noticeably in high-income countries in Europe, and in the United States. In these settings, survival was similar. Only 1 international study included patients diagnosed in middle-income countries (Belarus, Bulgaria, Montenegro, Romania, Serbia, Turkey, Ukraine). In this study (SEE consortium), the average 5-year survival for low grade astrocytic tumors was at least 15% lower than in more affluent countries (England, Germany, and the United States), but the gap in survival was smaller (approximately 10%) for high grade astrocytic tumors, for which little can be done (33).

Eleven out of 20 studies (55%) extended the use of ICCC to adolescents and young adults, using the broad definition “astrocytoma.” We were obliged to retain such definition in our analyses because these 11 studies did not stratify survival estimates by tumor subtype (eg, diffuse astrocytoma, glioblastoma) or WHO grade (ie, low grade and high grade), leading to a loss of precision and hampering the interpretation of results. The distribution of astrocytic tumors with different clinical behavior varies widely with age. Pilocytic astrocytoma, the most common nonmalignant astrocytic tumor, accounts for 60% of all astrocytic tumors in children compared with 47% in adolescents (15-19 years) and 19% in the 15- to 39-year-olds (34). Pilocytic astrocytoma is associated with a survival probability at 5 years of around 90%, whereas survival for higher grade astrocytoma is 50% or less (34). Therefore, any grouping strategy combining morphologies with very different outcomes will result in inflated, misleading survival estimates.

When we stratified the 11 studies using the information they provided on the eligible tumor behaviors, survival trends in adolescents (15-19 years) became slightly clearer, with lower 5-year survival when nonmalignant astrocytic tumors were excluded. When broader age groups were considered (upper age limit of 24 years or more), however, survival was similar after inclusion or exclusion of patients with nonmalignant astrocytic tumors. These tumors are rare in older adults, and their impact on survival estimates for the broad morphology “astrocytoma” is likely to be smaller with increasing age. Yet, in Eastern European adolescents, survival for all-behavior astrocytoma was in line with the values observed for malignant-only astrocytoma (18,19). Such a finding suggests underregistration of nonmalignant tumors.

We found that 5-year survival from astrocytoma in adolescents and young adults mostly varied between 48.0% and 71.0% during 1973-2004. Conversely, in children (0-14 years), survival from astrocytoma varied between 71.0% and 89.0% in most countries during 1970-2009 (37). Differences in survival between the 2 age groups, however, were less remarkable when more granular morphology definitions, instead of ICCC, were used, but studies adopting such strategy were scarce (37). Such discrepancy emphasizes the limitations of ICCC in accounting for the substantial differences in the morphology distribution of astrocytic tumors between children, and adolescents and young adults.

The classification proposed by Birch and Barr for tumors diagnosed in adolescents and young adults formed the basis of SEER AYA Site Recode (12–14), which implemented a bespoke classification scheme for adolescents and young adults in a large, population-based cancer registry. This classification scheme aimed to address the ICCC limitations by using WHO grade to further subdivide astrocytic tumors. We tried to regroup the studies based on SEER AYA Site Recode (Supplementary Figure 1, available online). Only 1 study adopted this classification, so we were obliged to mainly use the original morphology definitions (27). Five-year survival from low-grade astrocytic tumors was mostly in the range of 71.4%-93.4% during 1997-2007, and 5-year survival from high grade astrocytic tumors varied between 14.2% and 55.4% during 1991 and 2009. Survival for low-grade astrocytic tumors was remarkably higher than for anaplastic astrocytoma and glioblastoma, suggesting that SEER AYA Site Recode may be more appropriate than ICCC in describing survival in adolescents and young adults. The variation in survival within each morphology group, however, implies that combining different morphologies may still result in some loss of information. This seems particularly relevant to high grade morphologies, namely, anaplastic astrocytoma and glioblastoma. Anaplastic astrocytoma often recurs as glioblastoma, but outcomes at 5 years are remarkably different (Supplementary Figure 1, available online) (34). Further research is needed to understand whether SEER AYA Site Recode may replace ICCC in studies exploring survival disparities between children, and adolescents and young adults (12).

SEER AYA Site Recode also comprises the category astrocytoma NOS. In most of the studies using such definition, survival estimates were in the range of 55.0%-75.9% (7,26,33). These values are in line with those observed for diffuse astrocytoma (WHO grade II) (34). In ICD-O-3, diffuse astrocytoma is one of the alternative descriptors of astrocytoma NOS (11). Conversely, in the WHO classification (4th edition), astrocytoma NOS is not a separate definition and the corresponding ICD-O-3 code is attributed to diffuse astrocytoma (38). Given that the definitions “astrocytoma not otherwise specified” and “diffuse astrocytoma” refer to the same entity, we recommend against using the definition “astrocytoma NOS” as a synonym for unspecified astrocytic tumors (Supplementary Figure, available online).

Overall, there were no clear trends in 5-year survival from astrocytoma as a broad morphology group. In the United States, however, survival for adolescents (15-19 years of age) rose from 60.1% to 81.3% during the 2 decades between 1977 and 1997 (20). Conversely, survival for adolescents in Europe persisted in the range of 64.2%-66.0% during 1988-2001 (16,18,21). These findings may suggest that in the United States, outcomes for adolescents improved earlier than elsewhere, possibly due to the implementation of dedicated health services.

In adolescents and young adults, more than one-fourth of astrocytic tumors are glioblastomas (34). In 2005, a randomized clinical trial showed that 2-year survival was 26.5% in patients treated with radiotherapy plus temozolomide chemotherapy and only 10.4% in those receiving radiotherapy (39). The concomitant treatment has since become the standard of care for adults younger than 70 years. We could not explore the benefit of this treatment protocol at the population level, because very few survival estimates are available for patients diagnosed after 2005. In this systematic review, 5-year survival was in the range of 14.2%-23.1%. In older adults (40 years or older), 5-year survival is below 10%. Glioblastoma is defined as primary when it arises as a WHO grade IV lesion and secondary if it has developed from a lower grade glioma. Secondary glioblastomas are characterized by mutation of the isocitrate dehydrogenase (*IDH*) gene. Patients with *IDH*-mutated glioblastomas are younger than those affected by *IDH* wild-type glioblastomas (median age at diagnosis 32 years vs 59 years) and have a more favorable outcome (40). We chose to report only 5-year survival to improve comparability between studies, because it was the most commonly adopted outcome measure. For glioblastoma, however, shorter term survival estimates may be more informative.

Information on data quality indicators was inadequately reported and often totally missing (42% of studies). Data quality indicators tell us about cancer registry practices (eg, sources of data, type of follow-up) and affect the reliability of the data (41). Most frequently, studies indicated the proportion of microscopically verified tumors. Histologic confirmation of brain tumors may not be possible if the patient is clinically unfit for surgery or a biopsy, or if tumor location bars a diagnostic procedure. In each of the 2 large international studies reviewed here, the proportion of microscopically verified tumors varied widely between the participating registries. The average proportion of microscopically verified tumors in these 2 studies was similar (around 80%), but the SEE consortium also included middle-income countries, where access to care may be suboptimal (7,33). In some of the more affluent European countries, however, the proportion of microscopic verification was also rather low (eg, 63.3% in Italy) (7). Proportions that are very high may indicate overreliance on pathology reports and, therefore, a restricted number of data sources, leading to incompleteness of case ascertainment. Furthermore, patients with microscopically verified brain tumors may not necessarily represent the whole population, because they were at least able to undergo an invasive diagnostic procedure. If only these patients are included in the study, survival estimates may be higher. The overall completeness of case ascertainment was specified only in 4 studies, where it was 95% or more. Lower levels may suggest that a cancer registry fails to capture all the data within its catchment area, and this may lead to underascertainment of brain tumors (42).

Two-thirds of the studies estimated survival as all-cause survival (ie, observed survival). The cause of death is not used in international comparisons of cancer survival, because it may be unavailable or based on unreliable information from the death certificate (43–45). Observed survival can be readily obtained, but it is biased downward because it includes death from other causes (ie, background mortality). Background mortality varies between populations and over time, and it can be derived from life tables (46). Once competing risks of death are properly accounted for, the estimate will reflect net survival, which is the survival attributable exclusively to cancer. Net survival permits robust international comparisons, and it can now be directly estimated with the nonparametric, unbiased Pohar Perme estimator (10). Until recently, relative survival has been used as the best approximation of net survival, but this indicator does not allow for informative censoring. Informative censoring arises when the probability of dying from cancer is not independent of the probability of dying from other causes, and this is more frequent in the elderly. We have been obliged to compare studies that used different survival estimators, which may reduce the validity of some comparisons. Nevertheless, given that we considered relatively young patients and nearly all were diagnosed in affluent countries, failing to account for the rather low background mortality is unlikely to lead to substantial bias.

This systematic review presents some limitations. We adopted a wide range of morphology definitions to summarize survival variation in as much detail as possible. However, the number of survival estimates in some categories was small, hindering robust comparisons.

Many studies (75%) were partly or entirely based on regional data. We assumed that regional survival estimates applied to the whole country, but this assumption may not hold if provision of cancer care is unequally distributed. However, these regions were often also included in national or international studies. Estimates from studies with different geographical coverage did not differ substantially, so findings from smaller studies were fairly generalizable.

We were obliged to present trends using the central year of the calendar period covered by a given study regardless of its length. Although this strategy improved clarity, when 2 or more calendar periods overlapped, we could not use differences in length to explore nonlinear changes in survival. However, there were no substantial gains in survival for any of the morphologies, so comparisons referring to the central year may be acceptable.

In conclusion, there is a striking gap in knowledge about survival trends in middle-income and low-income countries, where disparities in access to care are reported (4). Studies with a wider scope extending to currently underrepresented geographical regions could fill this gap. Moreover, standardized data collection, data quality control, and data analysis using the same statistical methods are required to reduce heterogeneity and enable robust international comparisons. ICCC does not allow accurate reporting of survival from astrocytic tumors in adolescents and young adults because it does not account for the substantial differences in the histology distribution of brain tumors between adolescents and young adults, and children. Ultimately, ICCC should be revised. SEER AYA Site Recode, which proposes granular morphology groupings based on WHO grade, may inform such revision. Future studies should use improved classification systems to properly explore potential outcome disparities in adolescents and young adults and to inform cancer control plans.

## Funding

This work was supported by the Davidson and O’Gorman Fellowship from Children with Cancer UK.

## Notes

**Role of the funder:** The funder did not have a role in the design of the study; the collection, analysis, and interpretation of the data; the writing of the manuscript; and the decision to submit the manuscript for publication.

**Disclosures:** The authors declare that they have no competing interests.

**Author contributions:** FG, CA, and MPC: Conception and design. FG: Collection and assembly of data. All authors: Data analysis and interpretation. FG wrote the first draft, with input from CA and MPC: Manuscript writing. All authors: Final approval of manuscript. FG: Accountable for all aspects of the work.

## Supplementary Material

pkaa049_Supplementary_Data
