# Supplementary material for: Global Trends in Survival From Astrocytic Tumors in Adolescents and Young Adults: A Systematic Review
Source: JNCI Cancer Spectr. 2020 Jun 10;4(5):pkaa049. doi: 10.1093/jncics/pkaa049 (PMC7583144; doi:10.1093/jncics/pkaa049)

**Global trends in survival from astrocytic tumours in adolescents and young adults: a systematic review.**

Fabio Girardi MD, Claudia Allemani PhD, Prof Michel P Coleman FFPH

Cancer Survival Group, Non-communicable Disease Epidemiology Department, London School of Hygiene and Tropical Medicine

Supplementary Table 1. Strategy for searching Embase.

| Disease domain: central nervous system tumour | |
| --- | --- |
| #1 | “central nervous system tumo?r*".mp^1^. |
| #2 | "central nervous system cancer*".mp. |
| #3 | "central nervous system neoplasm*".mp. |
| #4 | "brain cancer*".mp. |
| #5 | "brain tumo?r*".mp. |
| #6 | "brain neoplasm*".mp. |
| #7 | "cns cancer*".mp. |
| #8 | "cns tumo?r*".mp. |
| #9 | exp^2^ central nervous system neoplasms |
| #10 | #1 or #2 or #3 or #4 or #5 or #6 or #7 or #8 or #9 |
| Outcome domain: survival | |
| #11 | "survival".mp. |
| #12 | “survival analysis”.mp. |
| #13 | exp survival rate |
| #14 | #11 or #12 or #13 |
| Study design domain: longitudinal observational studies | |
| #15 | "cancer registr*".mp. |
| #16 | “international comparison*”.mp. |
| #17 | exp epidemiology |
| #18 | exp life tables |
| #19 | exp registries |
| #20 | #15 or #16 or #17 or #18 or #19 |
| #21 | exp clinical trial, phase i or exp clinical trial, phase ii or exp clinical trial, phase iii or exp clinical trial, phase iv or exp controlled clinical trial or exp randomized controlled trial or exp double-blind method or exp random allocation or exp single-blind method |
| #22 | “randomized controlled trial”.mp. |
| #23 | "clinical trial".mp. |
| #24 | "clinical trial, phase i".pt^3^. |
| #25 | "clinical trial, phase ii".pt. |
| #26 | "clinical trial, phase iii".pt. |
| #27 | "clinical trial, phase iv".pt. |
| #28 | "controlled clinical trial".pt. |
| #29 | "randomized controlled trial".pt. |
| #30 | "clinical trial".pt. |
| #31 | #21 or #22 or #23 or #24 or #25 or #26 or #27 or #28 or #29 or #30 |
| #32 | #20 not #31 |
| Combination of the above domains | |
| #33 | #10 and #14 and #32 |
| #34 | "comment".pt. |
| #35 | "letter".pt. |
| #36 | "editorial".pt. |
| #37 | exp case reports |
| #38 | exp comment |
| #39 | exp letter |
| #40 | exp editorial |
| #41 | #34 or #35 or #36 or #37 or #38 or #39 or #40 |
| #42 | #33 not #41 |
| #43 | Animals |
| #44 | Humans |
| #45 | #43 not #44 |
| #46 | #42 not #45 |

^1^ mp: multi-purpose. The database looks for the keywords in the default set of fields.

^2^ exp: explosion. The database searches not only for the medical subject heading, but also for many related terms.

^3^ pt: publication type.

Question marks are used in systematic literature reviews to capture words with alternative spelling (e.g tumor and tumour).

Asterisks are used in systematic literature reviews to search for words with multiple endings (e.g. cancer and cancers; neoplasm and neoplasms).

Hashes are only used to improve reading.

Supplementary Table 2. Morphology groupings.

| All relevant definitions | |
| --- | --- |
| Definitions adopted in the studies | Definitions adopted the systematic review |
| Astrocytoma  Astrocytoma + pilocytic astrocytoma | Astrocytoma (broad group) |
| Astrocytoma low grade | Astrocytoma low grade |
| Astrocytoma NOS^1^  Astrocytoma NOS and other  Diffuse astrocytoma | Diffuse astrocytoma |
| Astrocytoma high grade  Glioblastoma and anaplastic astrocytoma | Astrocytoma high grade |
| Anaplastic astrocytoma | Anaplastic astrocytoma |
| Glioblastoma | Glioblastoma |
| SEER AYA^2^ Site Recode | |
| Definitions adopted in the studies | Definitions adopted the systematic review |
| Astrocytoma low grade  Diffuse astrocytoma | Specified low grade astrocytic tumours |
| Anaplastic astrocytoma  Astrocytoma high grade  Glioblastoma  Glioblastoma and anaplastic astrocytoma | Glioblastoma and anaplastic astrocytoma |
| Astrocytoma NOS  Astrocytoma NOS and other | Astrocytoma, NOS |
| Astrocytoma  Astrocytoma + pilocytic astrocytoma | Unclassified |

^1^ NOS: not otherwise specified

^2^ SEER AYA: Surveillance Epidemiology and End Results Adolescents and Young adults

Supplementary Table 3. Studies included in the systematic review.

| Author | Quality indicators | Estimator | 5-year survival |
| --- | --- | --- | --- |
| Aben et al., 2012 (22) | DCO^1^ excluded | Relative survival | Astrocytoma (male)*: 15-19 years: 51.1% (95% confidence interval 37.3-63.4%); 20-24 years: 53.7% (43.4-62.9%); 25-29 years: 55.6% (48.0-62.5%)  Astrocytoma (female)*: 15-19 years: 64.8% (49.0-76.8%); 20-24 years: 56.8% (45.5-66.7%); 25-29 years: 55.2% (46.2-63.4)  * Only malignant |
| Brodbelt et al., 2015 (30) | MV^2^: 90% | Relative survival | Glioblastoma: 20-44 years: 15.4% |
| Carreira et al., 2012 (23) | Not specified | Observed survival | Astrocytoma*: 15-19 years: 55.5% (95% CI 30.5-74.8%); 20-24 years: 81.3% (63.0-91.1%)  * Behaviour not specified |
| Desandes et al., 2007 (19) | Not specified | Observed survival | Astrocytoma: 15-19 years: 53.0% (95% CI 40.0-65.0%); 20-24 years: 57.0% (44.0-69.0%.) |
| Gatta et al., 2003 (16) | MV: 92%  DCO or autopsy with histology excluded  <5 years of follow-up: 0.7%  Unspecified morphologies: 6.5% | Observed survival | Astrocytoma: 66.0% (95% CI 62.4-69.4%) |
| Gatta et al., 2009 (21) | MV: 95%  DCO and autopsy only excluded  <5 years of follow-up: 2.6%  Unspecified morphologies: 3.8% | Observed survival | Astrocytoma: 1995-1999: Northern Europe: 51.6% (95% CI 39.6-63.6%), UK and Ireland: 48.6% (42.1-55.0%), Central Europe: 58.3% (49.1-67.5%), Southern Europe: 54.9% (45.8-64.0%), Eastern Europe: 65.0% (46.0-83.9%); 2000-2002: 55.8% (48.3-63.4%)  Astrocytoma (including pilocytic astrocytoma): 1995-1999: Northern Europe: 65.1% (55.8-74.4%), UK and Ireland: 57.9% (52.5-63.4%), Central Europe: 68.4% (61.1-75.7%), Southern Europe: 61.7% (53.8-69.6%), Eastern Europe: 66.0% (47.5-84.6%); 2002-2002: 64.2% (57.8-70.6%) |
| Georgakis et al., 2017 (33) | MV: 92% (SEER^3^); 71-85% (outliers: 57-96%)  DCO and lost to follow-up excluded  Unspecified morphology: 2.5-35% | Observed survival | Astrocytoma (low grade)*: SEE^4^: 59.0% (95% CI 54.0-63.0%); US: 76.0% (72.0-79.0%)  Glioblastoma and anaplastic astrocytoma: SEE: 28.0% (26.0-30.0%); US: 37.0% (35.0-39.0%)  Astrocytoma NOS^5^: SEE: 55.0% (52.0-57.0%); US: 72.0% (69.0-74.0%)  * Only malignant |
| Gondos et al., 2013 (26) | DCO or autopsy with histology excluded | Relative survival | Astrocytoma (low grade)*: SEER: 15-29 years: 89.1% (standard error 2.4%), 30-39 years: 72.6% (4.8%); Germany: 15-39 years: 76.0% (5.5%)  Glioblastoma and anaplastic astrocytoma: SEER: 15-29 years: 27.3% (3.5%), 30-39 years: 30.1% (2.6%); Germany: 15-29 years: 39.2% (4.5%), 30-39 years: 37.4% (3.4%)  Astrocytoma NOS: SEER: 15-29 years: 71.1% (4.3%), 30-39 years: 63.9% (4.3%); Germany: 15-29 years: 75.9% (5.6%), 30-39 years: 62.9% (5.1%)  * Only malignant |
| Ho et al., 2014 (28) | Not specified | Observed survival | Astrocytoma*: 18-40 years: 65.0% (95% CI 61.0-68.0%)  * Only malignant |
| Jung et al., 2012 (24) | MV: 100% | Observed survival | Astrocytoma*: 20-44 years: 59.4%  Anaplastic astrocytoma: 20-44 years: 39.6%  Glioblastoma: 20-44 years: 20.1%  * Only malignant |
| Linabery et al., 2008 (20) | MV: 95%  Lost to follow-up: 14% | Observed survival | Astrocytoma (adolescents): 1975-1979: 60.1% (95% CI 51.2-69.0%); 1985-1989: 73.1% (64.9-81.3%); 1995-1999: 81.3% (73.1-89.4%) |
| Narita et al., 2015 (31) | Not specified | Observed survival | Diffuse astrocytoma: 20-39 years: 83.2%*  Anaplastic astrocytoma: 20-39 years: 65.0%*  *Hospital-based estimates |
| Nicholson et al., 2013 (27) | Not specified | Observed survival | Astrocytoma (low grade)*: 87.0%  Astrocytoma (high grade): 18.0%  * Behaviour not specified |
| Ostrom et al., 2017 (34) | MV: 89% | Relative survival | Pilocytic astrocytoma: 93.4% (95% CI 91.6-94.9%)  Diffuse astrocytoma: 71.4% (69.1-73.6%)  Anaplastic astrocytoma: 55.4% (52.0-58.7%)  Glioblastoma: 23.1% (20.9-25.3%) |
| Pearce et al., 2005 (17) | Not specified | Observed survival | Astrocytoma: 1968-1977: 48.0% (95% CI 30.0-64.0%); 1978-1987: 50.0% (34.0-64.0%); 1988-1997: 60.0% (42.0-74.0%) |
| Smoll et al., 2014 (29) | Not specified | Relative survival | Anaplastic astrocytoma: 16-39 years: 50.4% (95% CI 47.0-53.6%) |
| Stiller et al., 2006 (18) | MV: 95-96%  DCO and zero survival excluded  Unspecified morphologies: 6-9% | Observed survival | Astrocytoma: Europe: 65.0% (95% CI 59.0-70.0%); British Isles: 71.0% (57.0-81.0%); Eastern Europe: 52.0% (37.0-65.0%); Northern Europe: 71.0% (59.0-80.0%); Southern Europe: 64.0% (54.0-73.0%); Western Europe: 64.0% (43.0-79.0%) |
| Thumma et al., 2012 (25) | Not specified | Observed survival | Glioblastoma: 20-29 years: 23.1% (95% CI 19.5-26.9%); 30-39 years: 16.7% (14.6-19.0%) |
| Trama et al., 2016 (32) | MV: 84-100%  DCO, autopsy only or zero survival excluded  Lost to follow-up: 0-9.7%  Unspecified morphologies: 0.0-18% | Relative survival | Astrocytoma: 15-19 years: 50.8% (standard error 2.5%); 20-24 years: 54.2% (2.2%); 25-29 years: 51.5% (1.7%); 30-34 years: 47.6% (1.4%); 35-39 years: 38.7% (1.2%)  * Only malignant |
| Visser et al., 2015 (7) | MV: 80%  Unspecified morphologies: 20% | Relative survival | Glioblastoma: 15-44 years: 14.2% (95% CI 13.0-15.5%)  Astrocytoma (NOS and other): 15-44 years: 56.1% (54.7-57.5%) |

^1^ DCO: death certificate only

^2^ MV: microscopic verification

^3^ SEER: Surveillance, Epidemiology and End Results

^4^ SEE: Southern and Eastern Europe consortium

^5^ NOS: not otherwise specified

Supplementary Figure. Five-year survival (%) from specified low grade astrocytic tumours, glioblastoma and anaplastic astrocytoma, and astrocytoma not otherwise specified (NOS) (Surveillance Epidemiology and End Results Adolescents and Young adults Site Recode). Error bars are 95% confidence intervals. For studies only providing point survival estimates, confidence intervals are shown as (survival estimate ± 1%).


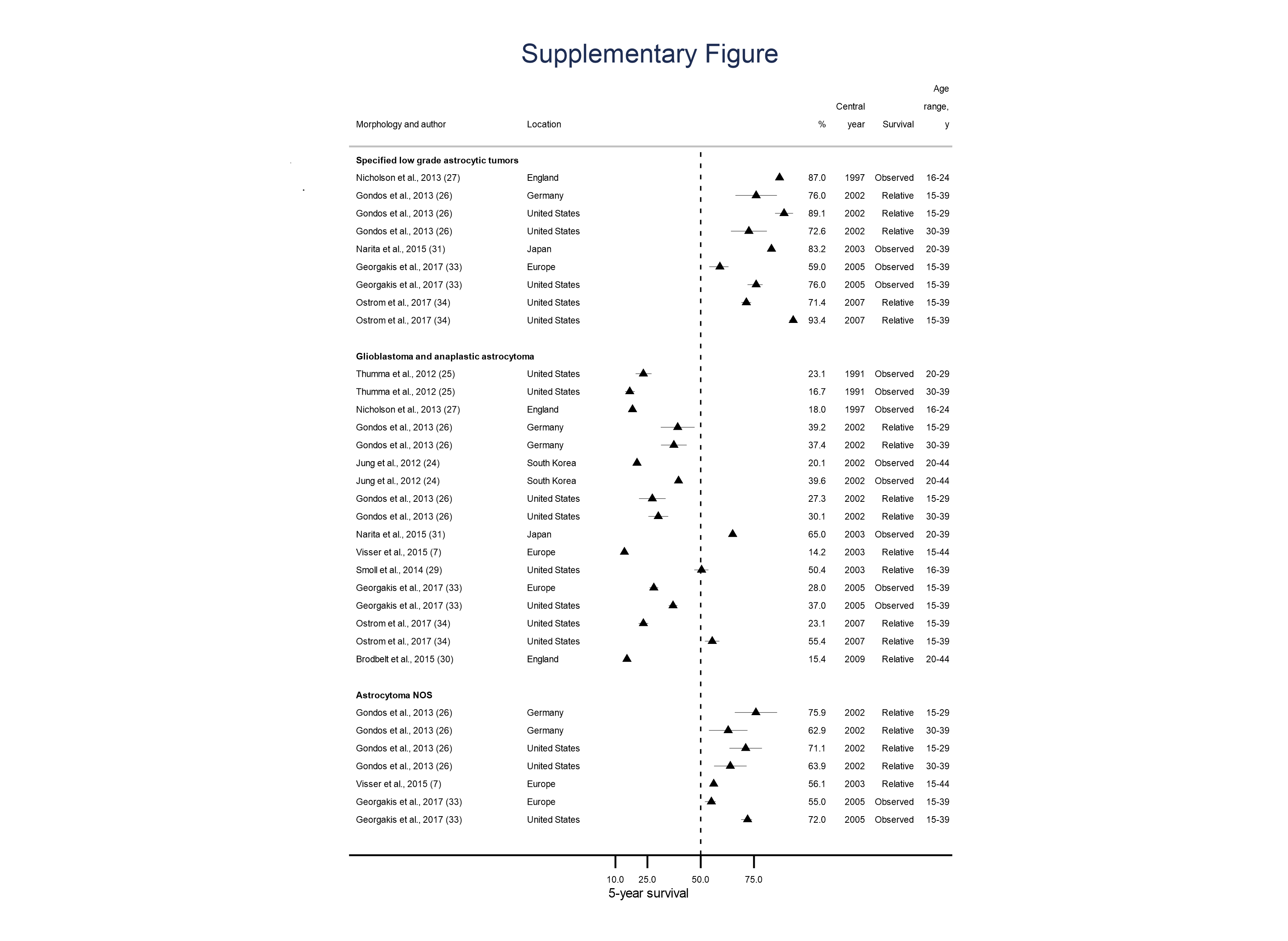

Supplement: pkaa049_Supplementary_Data [file pkaa049_supplementary_data.docx]
